# Perceptions and prevention practices on malaria among the indigenous Orang Asli community in Kelantan, Peninsular Malaysia

**DOI:** 10.1186/s12936-021-03741-y

**Published:** 2021-04-27

**Authors:** Mohd Bakhtiar Munajat, Mohd Amirul Fitri A. Rahim, Wathiqah Wahid, Mohd Ikhwan Mukmin Seri Rakna, Paul C. S. Divis, Sriwipa Chuangchaiya, Inke Nadia D. Lubis, Emelia Osman, Muhd Rafiq Mohd Kasri, Zulkarnain Md Idris

**Affiliations:** 1 Department of Parasitology and Medical Entomology, Faculty of Medicine, Universiti Kebangsaan Malaysia, 56000 Cheras, Kuala Lumpur, Malaysia; 2 District Health Office of Gua Musang, 18300 Gua Musang, Kelantan Malaysia; 3 Malaria Research Centre, Faculty of Medicine and Health Sciences, Universiti Malaysia Sarawak, 94300 Kota Samarahan, Sarawak Malaysia; 4 Department of Community Health, Faculty of Public Health, Kasetsart University, Chalermphrakiat Sakon Nakhon Province Campus, Sakon Nakhon, 47000 Thailand; 5 Department of Paediatric, Faculty of Medicine, Universitas Sumatera Utara, Medan, 20154 Indonesia

**Keywords:** Knowledge, attitude, and practice (KAP), Malaria, *Plasmodium knowlesi*, Indigenous population, Malaysia

## Abstract

**Background:**

Malaysia is on track towards malaria elimination. However, several cases of malaria still occur in the country. Contributing factors and communal aspects have noteworthy effects on any malaria elimination activities. Thus, assessing the community’s knowledge, attitudes and practices (KAP) towards malaria is essential. This study was performed to evaluate KAP regarding malaria among the indigenous people (i.e. Orang Asli) in Peninsular Malaysia.

**Methods:**

A household-based cross-sectional study was conducted in five remote villages (clusters) of Orang Asli located in the State of Kelantan, a central region of the country. Community members aged six years and above were interviewed. Demographic, socio-economic and KAP data on malaria were collected using a structured questionnaire and analysed using descriptive statistics.

**Results:**

Overall, 536 individuals from 208 households were interviewed. Household indoor residual spraying (IRS) coverage and bed net ownership were 100% and 89.2%, respectively. A majority of respondents used mosquito bed nets every night (95.1%), but only 50.2% were aware that bed nets were used to prevent malaria. Nevertheless, almost all of the respondents (97.9%) were aware that malaria is transmitted by mosquitoes. Regarding practice for managing malaria, the most common practice adopted by the respondents was seeking treatment at the health facilities (70.9%), followed by self-purchase of medication from a local shop (12.7%), seeking treatment from a traditional healer (10.5%) and self-healing (5.9%). Concerning potential zoonotic malaria, about half of the respondents (47.2%) reported seeing monkeys from their houses and 20.1% reported entering nearby forests within the last 6 months.

**Conclusion:**

This study found that most populations living in the villages have an acceptable level of knowledge and awareness about malaria. However, positive attitudes and practices concerning managing malaria require marked improvement.

## Background

Malaria, a febrile illness affecting people of all age groups, is a life-threatening parasitic disease with high morbidity and mortality worldwide. Approximately 230 million people are affected by malaria resulting in the deaths of 405,000 people globally in 2019 [1]. In Malaysia, significant progress towards the national elimination programme on human-only malaria has resulted in zero indigenous *Plasmodium falciparum* or *Plasmodium vivax* infections in 2018 [2]. Malaysia is among the 21 countries of the WHO E2020 group that are earmarked as ready for malaria elimination in 2020 [3]. Despite the success, within rural communities of Malaysia, the emergence of zoonotic transmission of the monkey malaria *Plasmodium knowlesi* has been less tractable to conventional malaria control and elimination programme [4, 5]. Nevertheless, the Malaysian government has reemphasized a great focus on malaria transmission foci at a local level as well as community understanding of malaria in order to achieve the elimination goal including the implementation of effective intervention strategies especially in remote communities of indigenous and tribal populations.

Orang Asli, the indigenous minority peoples of Peninsular Malaysia, comprises of three main tribes (i.e. Negrito, Senoi and Proto-Malay) with at least 18 distinct cultural-linguistic sub-tribes. They make up 0.6% of the total Malaysian population [6], with the Senoi tribe being the largest ethnic group constituting 55% of the total Orang Asli population with a large majority from the Temiar sub-tribe (Malaysia Department of Orang Asli). Since independence, the government of Malaysia has introduced various socioeconomic development programmes to improve the quality of life of the Orang Asli such as village resettlement, provision of electricity and water supply, construction of rural roads as well as easier access to education. Despite these proactive actions by the government, 37% of 869 Orang Asli villages throughout the country are still located in remote and forested areas [7]. Poverty and remote settlements exacerbate the health problems faced by these communities which include malnourishment and high incidences of infectious diseases [8–12]. Furthermore, the Orang Asli depend heavily on the surrounding forests, where they forage wild fruits, ornamental plants and wood products, and hunt wild animals as a source of income and food [13, 14]. These activities predispose them to mosquito bites and increase their risk for malaria infection.

In the past decade, studies conducted in Peninsular Malaysia have shown that malaria is common among the indigenous community of Orang Asli [10, 11]. This may be due to the low awareness or poor adherence among the Orang Asli towards malaria disease that might cause of lack of involvement in control activities. Therefore, it is crucial to inform and support the whole communities to continue or adopt preventive behaviours that can reduce the risk of infections [15]. Thus, this study aimed to evaluate the knowledge, attitudes and practices (KAP) regarding malaria among the Orang Asli, as they are one of the most vulnerable populations to malaria in Peninsular Malaysia. To date, this is the first study on KAP regarding malaria conducted in this community since the country declared zero indigenous malaria cases in 2018. The KAP identified could be used as baseline data to evaluate future tools and strategies formulated to control and/or eradicate malaria in the study area and other similar settings.

## Methods

### Ethics consideration

The study was approved by the Medical Ethics Committee of the National University of Malaysia (UKM) (Reference No. UKM PPI/111/8/JEP-2019-148) and the Department of Orang Asli Development or locally known as the Jabatan Kemajuan Orang Asli (JAKOA), Ministry of Rural and Regional Development Malaysia. Respondents were sensitized to the study objectives and procedures by the local health district personnel for the study participation.

### Study sites

**Fig. 1:**
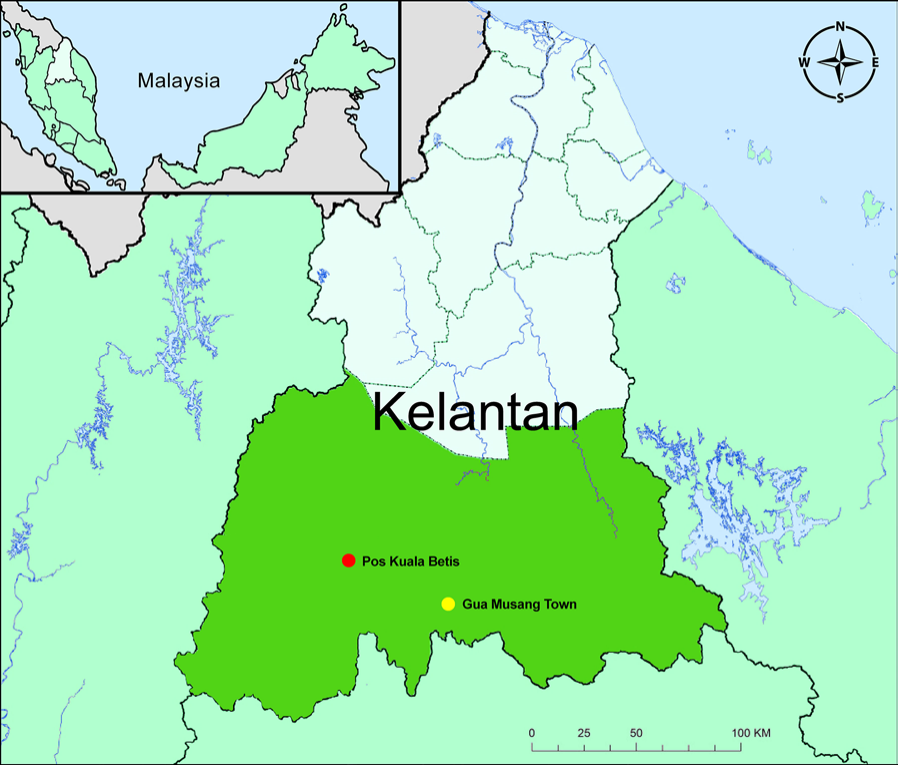
Map of Gua Musang District (dark green) in the State of Kelantan, Malaysia where surveillances were conducted. The study site in Pos Kuala Betis is located approximately 40 km from Gua Musang town

The study was conducted in Pos Kuala Betis (latitude 4^o^53′22′’N; longitude 101^o^45′30′’E), a cluster of five villages (Angkek, Betak, Sri Galas, Lambok and Podek) located at the Gua Musang district, Kelantan state, Peninsular Malaysia (Fig. 1). The Temiar sub-tribe of the Senoi was known to be the main indigenous Orang Asli in these villages. Located approximately 40 km from Gua Musang town, the typical climate of the study area is tropical monsoon with temperature ranged between 20 °C and 32 °C and average annual rainfall between 2000 mm and 4000 mm. The district has a consistent humidity of around 82% to 86%. All villages shared common environmental factors including adjacent to the river-basin and forest-fringe villages. While the main economic activity was centred on agriculture, such as palm oil plantation, the livelihood of the villagers mainly depended on rubber-tapping, labourers, farmers and gathering and selling forest products [13, 14].

An entomological survey conducted by the District Health Office of Gua Musang in August 2018 identified *Anopheles maculatus* as the main potential malaria vector, although all mosquitoes caught were negative for *Plasmodium* oocyst by quantitative PCR (Kelantan State Health Department, unpublished data). It has also been reported that this locality experienced recurrent malaria outbreaks within the past few years, which possibly related to autochthonous outbreaks of vivax malaria in the neighbouring state of Perak [8].

### Study design and data collection

The household-based descriptive cross-sectional study was carried out between June and July 2019. All villagers aged 6 years old and above were invited to take part in the study. The study protocol and consent were explained to the participants and their voluntary consent documented. Written informed consent was obtained from each adult participant at study registration. For the illiterate participant, written informed consent was obtained in the presence of an independent literate witness. For children and adolescents below 18 years old, written informed consent were obtained from parents or legal guardians. Despite the information on basic human health the children are getting from the school curriculum, their KAP towards prevention of vector-borne diseases particularly concerning malaria are still questionable and needs to be reviewed. District healthcare providers and community leaders (i.e. Tok Batin) were purposely involved in the study to facilitate participation and cooperation among the community. Participants were divided into three groups in accordance with age stratification practices of the JAKOA i.e. school-going children (6–18 years), young adults (19–40 years) and older adults (> 40 years).

A structured questionnaire was developed and administrated to each household and residents within the village cluster to gather information concerning demographics, socio-economics, knowledge, attitudes and prevention practices on malaria. The questionnaire included a mixture of closed-ended and open-ended questions. A pre-tested and validated pilot survey was conducted at communities from other districts to evaluate the clarity, adequacy and understandability of the questionnaire. Furthermore, preliminary interviews were also performed to ensure standard interviewers' method, knowledge, and suitable interview questions usage. To minimize the courtesy bias and improve the accuracy of participants’ self-reports, interviews were conducted by emphasizing anonymity by trained interviewers with on-site supervision. Before conducting the interviews, the lead researcher (ZMI) conducted a one-day training for the surveyors to explain each question in the questionnaire and how to conduct the interviews. Seven surveyors from the Department of Parasitology and Medical Entomology, Faculty of Medicine UKM and four local staffs from the District Health Office of Gua Musang visited each participant’s residence and conducted the face-to-face interviews using Malay language or local dialect.

### Data analysis

The data collected were tabulated into a spreadsheet of Excel (Microsoft, USA). Data analysis was performed using Stata/SE 13.1 for Windows (Stata Corp, USA). For categorical variables, data were presented as frequencies and percentages, while for continuous variables data were presented as mean ± standard deviation (SD). The differences between respondent’s characteristics were analysed using Chi-squared or Fisher’s exact tests. *P*-values of < 0.05 were considered statistically significant.

## Results

### Characteristics of respondents, households and malaria control measures

A total of 536 respondents and 208 households from Pos Kuala betis participated in this study (Table 1). Of the overall population (n = 941) based on the Demographic Surveillance System database of the local district health office, only 57% (n = 536) individuals from 208 households responded. The surveillance coverage varied among villages from 42 to 72%, being mostly female (60.3%) and the age of respondents ranged between 6 to 88 years old (mean [SD] age of 26.4 [17.1] years). Villagers from Angkek responded well in this study (72%) with a fair gender ratio distribution compared to other villages. The majority of the respondents were housewives (35.6%) and worked in the village (79.2%). Of the 208 surveyed households, most houses were constructed with wooden materials, such as walls (46.6%) and floors (7.1%), with corrugated iron roofs (91.8%).

**Table 1:** Socio-demographic data of respondents, households and malaria control measures in Pos Kuala Betis, Gua Musang District, Kelantan, Peninsular Malaysia in 2019

Characteristic	Overall	Angkek	Betak	Sri Galas	Lambok	Podek
Total number of surveyed households	208	65	17	40	55	31
Total population^a^, N	941	154	100	208	198	281
Total number of respondents, n (%)	536 (57)	111 (72)	42 (42)	100 (48)	132 (66.7)	151 (53.7)
Gender, n (%)						
Male	213 (39.7)	58 (52.3)	14 (33.3)	40 (40)	43 (32.6)	58 (38.4)
Female	323 (60.3)	53 (47.7)	28 (66.7)	60 (60)	89 (67.4)	93 (61.6)
Age, mean (SD), years	26.4 ± 17.1	26.1 ± 15.8	29 ± 18.3	27.4 ± 16.7	27.3 ± 18.5	24.5 ± 16.7
Age group, n (%), years						
6–18	220 (41)	44 (39.7)	17 (40.5)	38 (38)	53 (40.2)	68 (45)
19–40	212 (39.6)	49 (44.1)	14 (33.3)	38 (38)	54 (40.9)	57 (37.8)
> 40	104 (19.4)	18 (16.2)	11 (26.2)	24 (24)	25 (18.9)	26 (17.2)
Occupation^b^						
Housewife	123 (35.6)	22 (36)	7 (21.9)	34 (50)	39 (30)	21 (38.9)
Student	78 (22.6)	2 (3.3)	9 (28.1)	6 (8.8)	50 (38.5)	11 (20.4)
Agricultural sector	64 (18.6)	18 (29.5)	6 (18.8)	14 (20.6)	21 (16.2)	5 (9.3)
Labourer	49 (14.2)	17 (27.9)	7 (21.9)	4 (5.9)	8 (6.1)	13 (24)
Formal employment	19 (5.5)	2 (3.3)	2 (6.2)	8 (11.8)	3 (2.3)	4 (7.4)
Unemployed	12 (3.5)	0 (0)	1 (3.1)	2 (2.9)	9 (6.9)	0 (0)
Workplace^c^, n (%)						
Village	202 (79.2)	43 (72.9)	16 (72.7)	50 (83.3)	64 (90.1)	29 (67.4)
Town	53 (20.8)	16 (27.1)	6 (27.3)	10 (16.7)	7 (9.9)	14 (32.6)
Type of floors in the house^d^, n (%)						
Cement	83 (39.9)	32 (49.2)	4 (23.5)	22 (55)	9 (16.4)	16 (51.6)
Wood	98 (47.1)	21 (32.3)	11 (64.7)	17 (42.5)	40 (72.7)	9 (29)
Bamboo	27 (13.0)	12 (18.5)	2 (11.8)	1 (2.5)	6 (10.9)	6 (19.4)
Type of roofs of the house^d^, n (%)						
Thatch	17 (8.2)	12 (18.5)	0 (0)	0 (0)	1 (1.8)	4 (12.9)
Corrugated zinc	191 (91.8)	53 (81.5)	17 (100)	40 (100)	54 (98.2)	27 (87.1)
Type of walls in the house^d^, n (%)						
Cement	82 (39.4)	32 (49.2)	5 (29.4)	21 (52.5)	8 (14.5)	16 (51.6)
Wood	97 (46.6)	21 (32.3)	10 (58.8)	18 (45)	41 (74.5)	7 (22.6)
Bamboo	29 (14)	12 (18.5)	2 (11.8)	1 (2.5)	6 (10.9)	8 (25.8)
LLIN ownership, n (%)						
Yes	478 (89.2)	103 (92.8)	35 (83.3)	80 (80)	118 (89.4)	142 (94)
No	58 (10.8)	8 (7.2)	7 (16.7)	20 (20)	14 (10.6)	9 (6)
LLIN utilization/week, n (%)						
Every night	455 (95.2)	97 (94.2)	32 (91.4)	66 (82.5)	118 (100)	142 (100)
> 5 nights	2 (0.4)	0 (0)	2 (5.7)	0 (0)	0 (0)	0 (0)
3–5 nights	5 (1)	3 (2.9)	1 (2.9)	1 (1.2)	0 (0)	0 (0)
< 3 nights	16 (3.4)	3 (2.9)	0 (0)	13 (16.3)	0 (0)	0 (0)
IRS within the last 12 months, n (%)						
Yes	208 (100)	65 (100)	17 (100)	40 (100)	55 (100)	31 (100)
No	0 (0)	0 (0)	0 (0)	0 (0)	0 (0)	0 (0)

^a^Census from the Demographic Surveillance System of District Health Office Gua Musang

^b^Total of 345 respondents has no data on occupation

^c^Total of 245 respondents has no data on workplace

LLIN, Long-lasting insecticide bet net; IRS, Indoor residual spraying; SD, Standard deviation

With regard to malaria control measures, there was a remarkably high overall level of ownership of the long-lasting insecticidal net (LLIN) (89.2%) and compliance for nightly usage in the community (95.2%) (Table 1). Furthermore, indoor residual spraying (IRS) by the health authority was able to cover 100% of all surveyed households.

### Awareness of malaria and the prevention practices

Table 2 shows the age-stratification of respondents’ knowledge, attitudes and practices towards malaria. Overall, respondents from the age group of 19–40 years old had higher levels of overall correct knowledge on malaria compared to other age groups (*P* < 0.001). Slightly half of the total respondents were aware of the causes of malaria (51.9%) and able to identify the common symptoms (50.7%). Although many of them could relate fever with malaria (40.6%), only small proportions were able to determine rigor and chills as malaria symptoms (14.1%).

**Table 2:** Knowledge, attitude and practices (KAP) on malaria among different age groups of Orang Asli community in Pos Kuala Betis, Kelantan, Peninsular Malaysia in 2019

Variable	Total	Age group			*P*-value
		6–18	19–40	> 40	
Know the cause of malaria, n (%)					
Yes	278 (51.9)	48 (21.8)	162 (76.4)	68 (65.4)	< 0.001
No	258 (48.1)	172 (78.2)	50 (23.6)	36 (34.6)	
Cause of malaria, n (%)					
Mosquito bite	274 (98.6)	46 (95.8)	160 (98.8)	68 (100)	-
Stagnant water	2 (0.7)	1 (2.1)	1 (0.6)	0 (0)	
Dirty surrounding	1 (0.4)	0 (0)	1 (0.6)	0 (0)	
Poor nutrition	1 (0.4)	1 (2.1)	0 (0)	0 (0)	
Know symptom of malaria, n (%)					
Yes	272 (50.7)	52 (23.6)	149 (70.3)	71 (68.3)	< 0.001
No	264 (49.3)	168 (76.4)	63 (29.7)	33 (31.7)	
Symptoms of malaria*, n (%)					
Fever	257 (40.6)	49 (45)	145 (41.4)	65 (38.2)	0.859
Headache	91 (14.4)	16 (14.5)	48 (13.7)	27 (15.9)	
Rigor/chills	89 (14.1)	15 (13.8)	48 (13.7)	26 (15.3)	
Body ache	86 (13.6)	13 (11.9)	50 (14.3)	23 (13.5)	
Sweating	69 (10.9)	7 (6.4)	42 (12)	20 (11.8)	
Vomiting	27 (4.3)	6 (5.5)	14 (4)	7 (4.1)	
Nausea	8 (1.3)	3 (2.8)	3 (0.9)	2 (1.2)	
Know prevention of malaria, n (%)					
Yes	287 (53.5)	51 (23.2)	168 (79.2)	68 (54)	< 0.001
No	249 (46.5)	169 (76.8)	44 (20.8)	58 (46)	
Prevention of malaria^*^, n (%)					
Sleep under bed net	284 (50.2)	51 (54.3)	167 (49.3)	67 (50.4)	0.641
Spray insecticide at home/surrounding	108 (19.1)	13 (13.8)	67 (19.8)	28 (21.1)	
Take medication	80 (14.1)	11 (11.7)	48 (14.2)	21 (15.8)	
Wear protective clothing	30 (5.3)	8 (8.5)	19 (5.6)	3 (2.3)	
Burn mosquito coils	28 (4.9)	7 (7.4)	16 (4.7)	5 (3.8)	
Drain/add larvicide to stagnant water	20 (3.5)	2 (2.1)	12 (3.5)	6 (4.5)	
Wear insect repellent	15 (2.7)	2 (2.1)	10 (2.9)	3 (2.3)	
Know practice on managing malaria illness, n (%)					
Yes	280 (52.2)	49 (22.3)	160 (75.5)	71 (56.3)	< 0.001
No	256 (47.8)	171 (77.7)	52 (24.5)	55 (43.7)	
Practice on managing illness^*^, n (%)					
Go to the clinic immediately	273 (70.9)	47 (61)	156 (71.2)	70 (78.7)	0.152
Purchase medication from local shop	49 (12.7)	14 (18.2)	25 (11.4)	10 (11.2)	
Seek treatment from a traditional healer	40 (10.4)	8 (10.4)	25 (11.4)	7 (7.9)	
Wait out the symptoms until well	23 (6)	8 (10.4)	13 (5.9)	2 (2.2)	

^*^Some respondents with a combination of more than one answer

Almost all respondents (98.6%) acknowledged mosquitoes as the main cause of malaria transmission. However, only 53.5% of them have adequate knowledge of the malaria preventive measures with significantly higher (*P* < 0.001) in the 19–40 years group than their counterparts. Respondents from all age groups also agreed that the use of bed nets would be the best measure to prevent malaria (50.2%), however, the observations between age groups were not significant. Furthermore, a slight majority (52.2%) of the respondents reported knowing on the practice of managing illness especially among the older age groups (*P* < 0.001). The respondents also showed high levels of seeking treatment at the government clinics (70.9%). Only a small proportion was still considered an alternative treatment, such as practising traditional medicine (10.4%).

### Contributing factors of zoonotic malaria transmission

Almost all respondents (94.8%; Table 3) in all villages reported spending the nights at home (*P* = 0.001). Furthermore, almost half of the respondents (47.2%) mentioned that they have seen monkeys within 500 m from their houses. However, most of this observation was reported by villagers from Sri Galas and Lambok (*P* < 0.001). In contrast, although the villages are located near to a forested area, only 20% of the respondents had entered the nearby forest within the last 6 months, especially respondents from Angkek compared to other villages (*P* < 0.001).

**Table 3:** Contributing factors of zoonotic malaria transmission among Orang Asli community in Pos Kuala Betis, Kelantan, Peninsular Malaysia in 2019

Characteristic	Overall	Angkek	Betak	Sri Galas	Lambok	Podek	*P*-value
Frequency of spending the night at home, n (%)							
Every night	508 (94.8)	97 (87.4)	41 (97.6)	95 (95)	126 (95.5)	149 (98.7)	0.001
Occasionally	28 (5.5)	14 (12.6)	1 (2.4)	5 (5)	6 (4.5)	2 (1.3)	
Monkey presence within 500 m from the house, n (%)							
Yes	253 (47.2)	53 (47.7)	2 (4.8)	98 (98)	97 (73.5)	3 (2)	< 0.001
No	283 (52.8)	58 (52.3)	40 (95.2)	2 (2)	35 (26.5)	148 (98)	
Enter the nearby forest within the last 6 months, n (%)							
Yes	108 (20.1)	49 (45.4)	8 (19)	15 (15)	17 (12.9)	19 (12.6)	< 0.001
No	428 (79.9)	62 (54.6)	34 (81)	85 (85)	115 (87.1)	132 (87.4)	

## Discussion

This study was designed to assess the indigenous community with regards to the knowledge, attitudes and preventive practices on malaria, focusing on the remote indigenous settlement of Orang Asli located in the interior part of Peninsular Malaysia. Subsequently, high-risk behaviour and living conditions were also observed to identify any potential factors that could influence zoonotic malaria transmission in the community. The indigenous community normally lives in deprived environments with an inadequate intake of energy, has a poor nutritional status and expose to high parasitic infections [13, 16–18]. Traditionally leading a hunter-gatherer lifestyle in a tropical jungle environment, these indigenous people are also routinely exposed to malaria [8, 19].

In this study, almost all of the respondent acknowledges mosquito bite as the main transmission of malaria infection. These are encouraging results when compared to only 50% of indigenous people who made a correct association in the past decade [9]. Recurrent visits by the health personnel from the Malaysia Ministry of Health to apply IRS to the respondent’s home and providing as well as updating records about their LLIN physical condition creates a personal and informal awareness education. The important awareness about malaria vectors, the pattern of mosquito behaviour (biting and resting times) and breeding sites have been linked with the severity of malaria in remote communities [20]. One study in Tanzania showed a poor understanding of the vectorial capacity of the mosquito to transmit malaria has led to a high prevalence of the disease in the area [21].

Despite the very high numbers of respondents utilizing LLIN every night in this study, about half reported understanding the benefit of bed net as a prevention tool against malaria. It is not clear what could be driving the low understanding among the community, however, lack of knowledge, perceptions of risk and inadequate social behaviour change communication programme may contributing to the observed pattern [22, 23]. This study also noted that a small number of respondents stated that they did not use bed nets because of low mosquito population density and low disease incidence. Since the majority of the respondents (i.e. 95%) in this study remain inside their houses at night time, improving ownership and utilization of bed nets could positively impact transmission risk in the village despite *An. maculatus* mosquito vectors in the area typically showing an exophagic behaviour. The bed net policy in Malaysia is to annually replenish the old or damaged nets and distribute them house-to-house so that malaria prevention campaigns continue to prevent outbreaks [24]. Given the changing malaria situation in the country, continued efforts are needed to emphasize the benefits of operational vector control activities for eliminating any localized residual foci of transmission. For wide coverage of bed net use through equitable access among the Orang Asli population, various stakeholders including malaria control programme managers at the state and district levels should include provisions for subsidies to create sustained availability, affordability and increased use of bed nets. Furthermore, reasons for poor bed nets utilization among some of the Orang Asli respondents need to be explored through qualitative research.

Analysis on the practice for the treatment-seeking behaviour of the respondent showed that the majority prefer to seek clinical treatment for malaria. Direct observation of the studied area also revealed that the community has easy access to nearby facilities and free medical services provided by government-run healthcare facilities. This suggests a good availability of health services and the accessibility of healthcare facilities in the study region [25]. However, a small proportion of respondents still practice self-medication procured from the nearby sundry shop as well as observed traditional healing methods. There are multiple factors which may positively or negatively influence the practice for that treatment-seeking behaviour of individual or communities. A study by Matsumoto-Takahashi et al*.* revealed that malaria patients treatment-seeking behaviour has been linked with factors, such as age, gender, educational status, financial resources, access to health care facilities, the severity of the disease, cultural beliefs and practices about the cause and cure of the illness [26]. Furthermore, a study conducted in Nepal showed that participants preferentially consulted traditional healers suggesting a lack of appropriate facilities and awareness in that region [27]. In the case of Orang Asli, they often associate diseases with ghosts and evil spirits and people normally consulted their local sorcerers who provided rituals and remedies to fight the evil spirits [9].

This study also uncovered that the presence of monkeys within the villages is an important potential risk factor for zoonotic malaria transmission. The probability of introduced cases of the simian malaria parasite in areas where monkeys live near human settlements would be highly likely. As indigenous populations residing primarily within natural jungle habitats and typically practising agro-subsistence nomadic lifestyle, their communities are always at risk of persistence exposure to zoonotic malaria as they come into close contact with macaques or other monkey species, which could harbour simian *Plasmodium* species particularly *P. knowlesi* and *Plasmodium cynomolgi* [28–34]*.* A recent report of *P. knowlesi* isolated from patients from Gua Musang showed that this parasite is genetically distinguished from those of the Malaysian Borneo, indicating the different evolutionary history of this parasite [35]. Furthermore, the availability of *An. maculatus* as a competent vector in the area completes the transmission cycles and potentially able to transmit the parasite to the community. Even though being classifies as anthropophilic, *An*. *Maculatus *has been known to feed on monkeys when available and shown to be susceptible to simian malaria parasites in laboratory settings [36].

A number of caveats should be considered in this study. First, while the convenience household sampling approach used in this study was efficient and cost-effective, it has an inherent selection bias. The survey was conducted during the weekdays, meaning that children and adolescents of school ages (i.e. 6–18 years) were disproportionally represented. Second, the survey underreported adult males that could have led to an underestimation of overall data. Many adult males in the community are engaged in work activities during daytime hours when the cross-sectional survey was held, they were either be in the farm or nearby forest. The malaria KAP status in this mobile ad hard-to-reach group is not well characterized and warrant further investigation. Third, the villages in this study were selected based on the historical and unpublished records of malaria cases, the distance to the urban area, and the accessibility of field staff. The results of this study cannot be extrapolated to the general population. Nevertheless, the results can be useful to exhibit the problems around malaria KAP in the indigenous and non-indigenous people.

## Conclusion

The study finding disclosed a high and acceptable level of knowledge about malaria as well as a positive attitude towards malaria control interventions accompanied by promising malaria prevention and treatment-seeking behaviour among Orang Asli in Kelantan, Peninsular Malaysia. It has furthermore shown a good engagement and commitment between the indigenous community and the health authority in reassuring that the continued efforts to eliminate malaria in the country. Although knowledge regarding malaria among Orang Asli communities was high, a substantial proportion of them will require more information concerning the treatment-seeking behaviour and potential risk of zoonotic malaria infection. This information can be channelled through the local healthcare personnel that already involved in providing basic knowledge of malaria to ensure the message to the community is accurate and appropriate. Furthermore, continued malaria surveillance, vector control including IRS and LLIN, education and information campaigns are important not only among the Orang Asli community but other vulnerable communities in the country.

## Data Availability

The dataset used and/or analysed during the current study are available from the corresponding authors upon request.

## Abbreviations

IRS: Indoor residual spraying
JAKOA: Department of Orang Asli Development
KAP: Knowledge, attitude and practice
LLIN: Long-lasting insecticidal nets
WHO: World Health Organization
